# Fano-resonant hybrid Metasurface for Carbon Dioxide sensing at telecommunication wavelengths

**DOI:** 10.1038/s41598-026-53746-3

**Published:** 2026-05-25

**Authors:** Norhan A. Salama, Mohamed A. Swillam

**Affiliations:** Department of Physics, School of Science and Engineering, The American University in Cairo, Cairo, 11835 Egypt

**Keywords:** Engineering, Materials science, Nanoscience and technology, Optics and photonics

## Abstract

The development of compact and selective carbon dioxide (CO₂) sensors is in significant demand due to the severe effect of CO₂ on global warming and human health. Introducing a functional smart material for CO_2_ adsorption represents an excellent solution for miniaturized selective devices. However, most of these devices operate beyond the telecommunication wavelengths, limiting their applicability for photonic integrated circuits (PICs). In this work, we report a hybrid metasurface – polyhexamethylene biguanide (PHMB) design for a highly selective and sensitive optical sensor. We leverage the Fano-resonant metasurface of coupled nanodisk and nanobar resonators to be integrated with infiltrated polymer (PHMB) as a functional smart material for selective CO_2_ adsorption. The design parameters are optimized at an operational wavelength of 1.55 μm to achieve an optimal trade-off between Q-factor and modulation depth. Our proposed design offers an outstanding sensitivity reaching an of 45 pm/ppm (212 nm/RIU) accompanied by an exceptional Q-factor of approximately 8$\:{\times\:10}^{4}$. Furthermore, we demonstrate that increasing the polymer thickness remarkably enhances the sensitivity up to 312 nm/RIU, while the figure-of-merit (FOM) reaches 12,500. These results hold significant promise for the development of highly selective, sensitive and miniaturized sensors with ease of fabrication and low cost.

## Introduction

The greenhouse effect resulting from excessive emission of carbon dioxide (CO₂) — now reaching concentrations exceeding 400 ppm — represents a critical global threat. Exposure to elevated CO₂ levels has been associated with serious health risks such as sick building syndrome, cardiovascular diseases, respiratory illnesses, and even cancer. Moreover, CO₂ concentration in food packaging serves as an important indicator of food freshness and safety within the ecosystem. These considerations highlight the strong demand for developing high-efficiency, low-cost CO₂ gas sensors. The non**-**dispersive infrared (NDIR) sensor has been the primary technology for gas detection, as it exploits the characteristic infrared absorption fingerprints of gases. However, NDIR systems require a bulky absorption cell to achieve sufficient light–gas interaction, which limits their practicality for compact applications^1^. Surface plasmon resonance (SPR) sensors utilizing the Kretschmann configuration have also been widely adopted; however, they often suffer from loss-related constraints^2^. Optical fiber-based sensors integrated with color-changing dyes offer compactness, low cost, and high sensitivity, but their stability is compromised by photobleaching, which degrades performance over time^3^.

Moreover, the aforementioned approaches are not suitable for photonic integrated circuits (PICs), as they mostly operate beyond telecommunication wavelengths. The telecommunication wavelengths fall within the range between 1260 and 1650 nm. This is the standard range of data center communication and optical telecommunication specifically at 1310 and 1550 nm^4^. The 1.55 μm band is the centerpiece of PIC especially in silicon on insulator devices and optical fibers. Design of PIC mainly relies on integrated optical components including laser sources, photodetectors, active modulators, active beam steering, dynamic hologram rendition and sensors^5–7^. These components are designed to work at the operational wavelength of 1.55 μm. At 1.55 μm, silicon demonstrates optical linearity and full transparency. These properties are essential for sensing applications. In contrast, at lower wavelengths silicon may introduce two-photon absorption^8^. In addition, the sensing material such as polyhexamethylene biguanide (PHMB), the scope of this study, is transparent at this wavelength. Unlike previous PHMB-based sensors working at 1.33 μm and 1.04 μm, the choice of 1.55 μm wavelength of operation sidesteps the two-photon absorption in silicon and is compatible with PIC.

Various photonic approaches such as plasmonic-heterojunction nanostructures^9^, metal-insulator-metal (MIM)^10,11^, Photonic crystals^12^, plasmonic photonic crystal fiber^13,14^, and Metasurfaces^15^ have emerged as fast-growing technology platforms that push the boundaries of sensitivity, compactness, and CMOS compatibility. These technologies offer exceptional optical properties specifically the sharp spectral lines that are essential for sensing applications. Plasmonic devices including plasmonic–heterojunction nanostructures, MIM, plasmonic photonic crystal and plasmonic metasurfaces advance the sensitivity through enhancing light-matter interaction. MIM structures leverage Fano resonance for refractive index sensing^10^. Photonic crystal fiber (PCF) has been proposed as a temperature sensor that utilizes a flat, metal-coated trapezoidal surface^14^. Photonic crystals offer uniquely guided modes^12^. Metasurfaces consist of planar arrays of periodic nanoantennas, enabling exceptional control over light propagation. They are generally classified into two main classes: plasmonic and dielectric metasurfaces. Plasmonic metasurfaces generate a localized surface plasmon resonance (LSPR) offering high sensitivity to the surrounding medium. For instance, a metasurface biosensor comprising an array of gold bowtie, functionalized with gold nanoparticles and antibodies of epidermal growth factor receptor (EGFR) has been proposed for detection of EGFR in terahertz region^16^. Split ring resonator sensor (SRR) is proposed for detecting low concentration bacteriophage viruses with sizes ranging from 60 nm to 30 nm^17^. A dielectric layer sandwiched between a metallic nanobar array and a thin Ag film exhibits multiple absorption bands across near- and mid-IR region, with peak absorbance exceeding 96%^18^. A gold (Au) cross-bar-patch structure, and a polyimide dielectric spacing layer located on an Au grounded plane metasurface offer multiple absorption bands across terahertz range with sensitivities reaching up to 14 THz/RIU^19^. For a similar design with three crossbars, the spectrum becomes continuous over a bandwidth of 0.90 THz^20^. In addition, plasmonic metasurfaces incorporating graphene add further advantage of tunable optical conductivity and wavelength. For example, a structure comprising SiO_2_/Ag/SiO_2_/Ti/SiO_2_ stack with integrated monolayer graphene sheets, featuring a dual bowtie aperture pattern exhibits multiple tunable modes across the wavelength range between 0.4 and 1.200 μm enabling strong field confinement and energy harvesting^21^. A broadband terahertz metamaterial absorber through bi‑layer hybridization of metal and graphene offers almost perfect absorption and tunability along a terahertz range^22^. However, plasmonic metasurfaces suffer from energy dissipation as heat in metallic structures.

Alternatively, dielectric metasurfaces are a sensing approach overcoming the significant losses inherent in plasmonic structures. For example, all-dielectric sensor based on a periodic array of silicon (Si) plates with square nanoholes shows a remarkable sensitivity with reduced energy losses^23^. A metasurface based on a symmetry-broken rectangular lattice of silicon nanodisk dimers on a SiO_2_ substrate exhibits a sharp quasi-bound states in the continuum resonance^24^.

Although metasurfaces offer strong promise for high sensitivity, selectivity and cost efficiency, gas sensing is particularly challenging for many reasons. First, gas sensing requires a sufficiently long optical path length for achieving light–gas interaction. Second, gases possess very close refractive indices, adding a limitation of selectivity toward certain gas molecules. Third, the difficulty in detecting the small refractive index changes induced by gas molecules imposes further limitation. Finally, designing a sensor device while ensuring repeatability and long-term stability of the sensor performance is also challenging.

In light of that, the scientific community has devoted special attention to overcome the aforementioned obstacles. For instance, Lochbaum et al. developed special gas chamber design based on spherical geometry, enabling multiple reflections of optical rays for enhanced optical path length, and so that gas-light interaction^25^. Salama et al. proposed a metasurface design that is optimized to match the spectral fingerprint of the gas molecules. This matching accompanied by high Q-factor enhances the spectral response to the abrupt change of the refractive index at the gas’s fingerprint^26–28^. However, the design does not address the constraints of the poor gas-light interaction. Alternatively, a functional CO_2_ adsorbing material, such as polymeric coatings that are capable of selectively adsorbing CO₂ molecules while maintaining reversible reactions, represents a strong promise for CO_2_ sensing. Polymers such as polyethyleneimine/poly(vinyl alcohol) and polyhexamethylene biguanide (PHMB) films have shown high potential for developing stable, compact, and reusable CO₂ sensors^29–32^. This approach has been used in a number of sensor designs including; Silicon nanocrystalline meta-atom periodically arranged on a thin gold layer^32^, Au-disk/TiO2-cylinder/Au-film metasurface^33^, and cross wire of Mo metasurfaces pattern^34^. Despite the fact that these designs offer high CO_2_ selectivity, the metallic inclusions impose significant losses, that limit the design performance.

In this work, we leverage the Fano resonant metasurface of coupled silicon nanodisk and nanobar resonators and integrate it with infiltrated PHMB polymer for sensor design. Initially, we use the PHMB thickness of t = 220 nm– the same thickness as the coupled resonators. We tailor the design parameters to work within the telecommunication wavelengths (~ 1.55 μm). The choice of this wavelength is motivated by the compatibility with photonic integrated circuits (PIC). At 1.55 μm, both PHMB and CO₂ exhibit negligible spectral absorption. PHMB instead plays a vital role in electromagnetic field confinement within its layer. This offers strong promise for enhancing the refractometric sensitivity even with a minor refractive index change due to CO₂ adsorption. In contrast, the mid-IR regime offers stronger CO₂ absorption fingerprints, which can be leveraged in loss-coupled sensing mechanisms for potentially higher sensitivity^26^. However, PHMB possesses its own vibrational modes across the mid-IR, which could introduce parasitic absorption losses. Additionally, the SiO₂ substrate introduces phonon absorption losses at longer wavelengths. Therefore, the choice of 1.55 μm is appropriate for designing a low-cost, photonic-integrated sensor with a non-interfering optical response. Secondly, we interpret the coupling mechanism between the resonators in presence of PHMB layer, through investigating the electric field profiles across the three metasurface designs – nanodisk only, nanobar only and coupled nanodisk and nanobar. Thirdly, we optimize the gap distance (g_1_) between the resonators to achieve the highest Q-factor with a good modulation depth. Fourthly, we investigate the sensitivity performance of the design. Finally, we investigate the effect of different PHMB thicknesses on sensitivity performance. The proposed design demonstrates an outstanding Q-factor reaching up to 8$\:{\times\:10}^{4}$accompanied by remarkable sensitivity 45 pm/ppm- 212 nm/RIU and Figure-Of-Merit (FOM) of 12,500 RIU^− 1^ and limit of detection (LOD) down to $\:8\times\:{10}^{-5}$. To the best of our knowledge, this is the first metasurface–PHMB platform operating at the telecommunication wavelength for highly selective CO₂ sensing.

## Sensitivity performance metrics

Refractometric optical sensors are designed to provide sharp spectral resonances that can be shifted in response to changes in the surrounding medium. Sensor performance is evaluated using key sensitivity metrics, including quality factor (Q-factor), sensitivity (S), figure of merit (FOM) and limit-of-detection (LOD). The Q-factor is a crucial parameter that quantifies the sharpness of the spectral resonance through its full width at half maximum (FWHM). The Q-factor is of pivotal importance for spectral selectivity and is defined as:

1 $$\:\mathrm{Q}=\frac{{\lambda\:}_{0}}{FWHM}$$

where λ_0_ is the resonance wavelength. Sensitivity (S) describes the spectral shift induced by a change in the refractive index of the surrounding medium ($\:\varDelta\:n),$ which corresponds to a change in the concentration of the measured analyte. S is the primary performance metric and is calculated with respect to the refractive index as:

2 $$\:S=\frac{\varDelta\:{\lambda\:}_{0}}{\varDelta\:n}$$

Alternatively, it can be calculated with respect to the change in the analyte concentration as:

2’ $$\:S=\frac{\varDelta\:{\lambda\:}_{0}}{\varDelta\:c}$$

The figure of merit (FOM) is a metric that quantifies the overall sensor performance by relating the two metrics — Q-factor and sensitivity. FOM is defined as the ratio between sensitivity and the FWHM:

3 $$\:FOM=\frac{S}{FWHM}$$

The limit of detection (LOD) represents the minimum detectable change in the refractive index and is calculated as follows:

4 $$\:\mathrm{L}\mathrm{O}\mathrm{D}\:=\frac{FWHM}{S}$$

### Metasurface design and simulation method

**Fig. 1:**
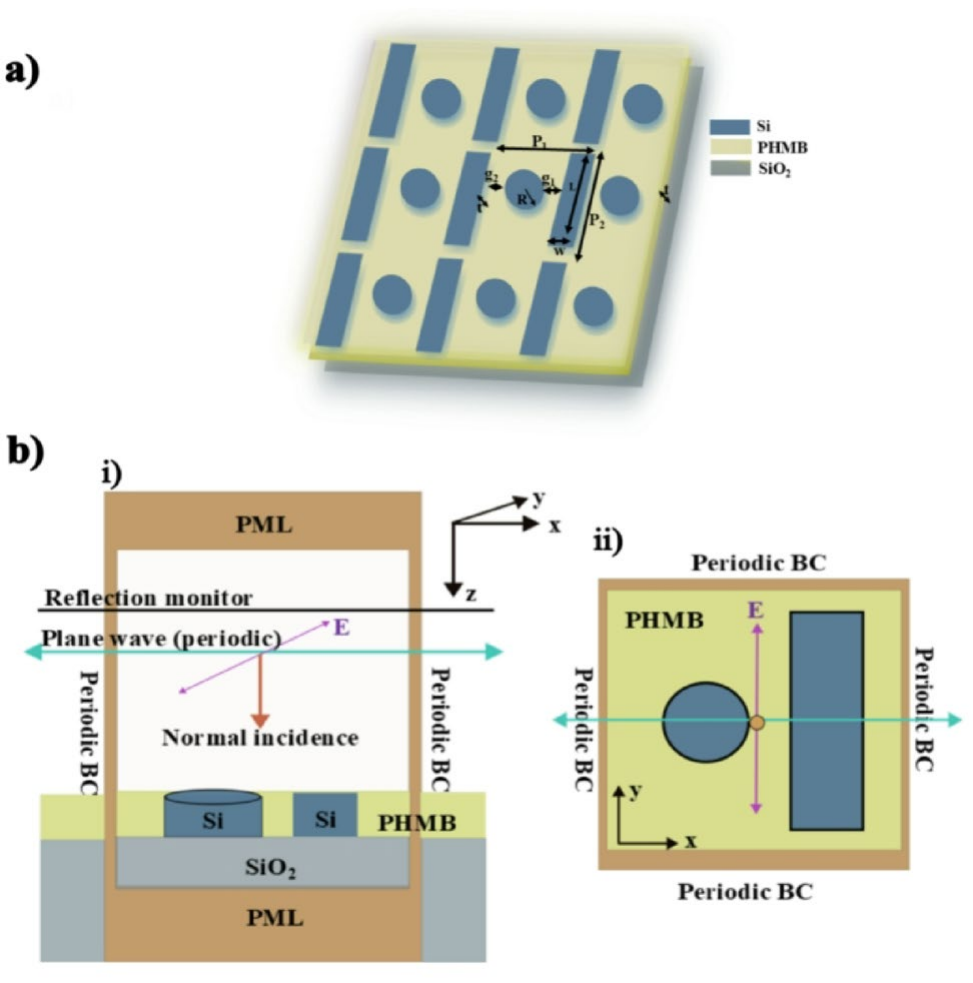
Schematic and simulation setup of the hybrid metasurface design: **(a)** the complete metasurface structure with parameters *R* = 195 nm, w = 250 nm and L = 870 nm, g_2_=150 nm and P_2_ = 1,020 nm, while g_1_ and P_1_ are varied for optimization, **(b)** Lumerical three-dimensional simulation setup in (i) the xz-plane and (ii) the xy-plane.

The schematic of the proposed hybrid metasurface design consisting of periodic unit cells mounted on a SiO_2_ substrate is presented in Fig. 1a. Each unit cell comprises coupled nanodisk and nanobar resonators integrated with infiltrated PHMB polymer.

filling the space between the resonators with a thickness of (t = 220 nm). The geometrical parameters of the structure are defined as follows: nanodisk radius of (*R* = 195 nm), nanobar width of (w = 250 nm) and length of (L = 870 nm), the first gap distance between the resonators (g_1_) and the corresponding horizontal pitch (P_1_) are varied for optimization, while the second gap is fixed at (g_2_=150 nm), and the vertical pitch of the unit cell is of (P_2_ = 1,020 nm). We numerically investigate the optical response of the design using the finite-difference time-domain method (FDTD) implemented in the commercial software—Ansys Lumerical^35^. We use a three-dimensional simulation setup for one unit cell with periodic boundary conditions in the x- and y-directions (Fig. 1b, ii) and perfectly matched layers (PML) in the z-direction (Fig. 1b, i). A periodic plane-wave source is positioned one wavelength above the metasurface, while the polarization is oriented along the y-direction, which corresponds to the long axis of the nanobar. The reflection monitor is placed above the source to record the reflection spectra. The refractive index of PHMB at very low concentrations of CO_2_ is taken as (*n* = 1.54). The dispersive refractive indices of Si and SiO_2_ are obtained from the built-in material library of Ansys Lumerical. We use an adaptive auto-nonuniform mesh refinement level of 5, allowing graded meshing near surfaces with high refractive index. This type of meshing provides high accuracy and reliable simulation results.

### The optical response of the proposed sensor

**Fig. 2:**
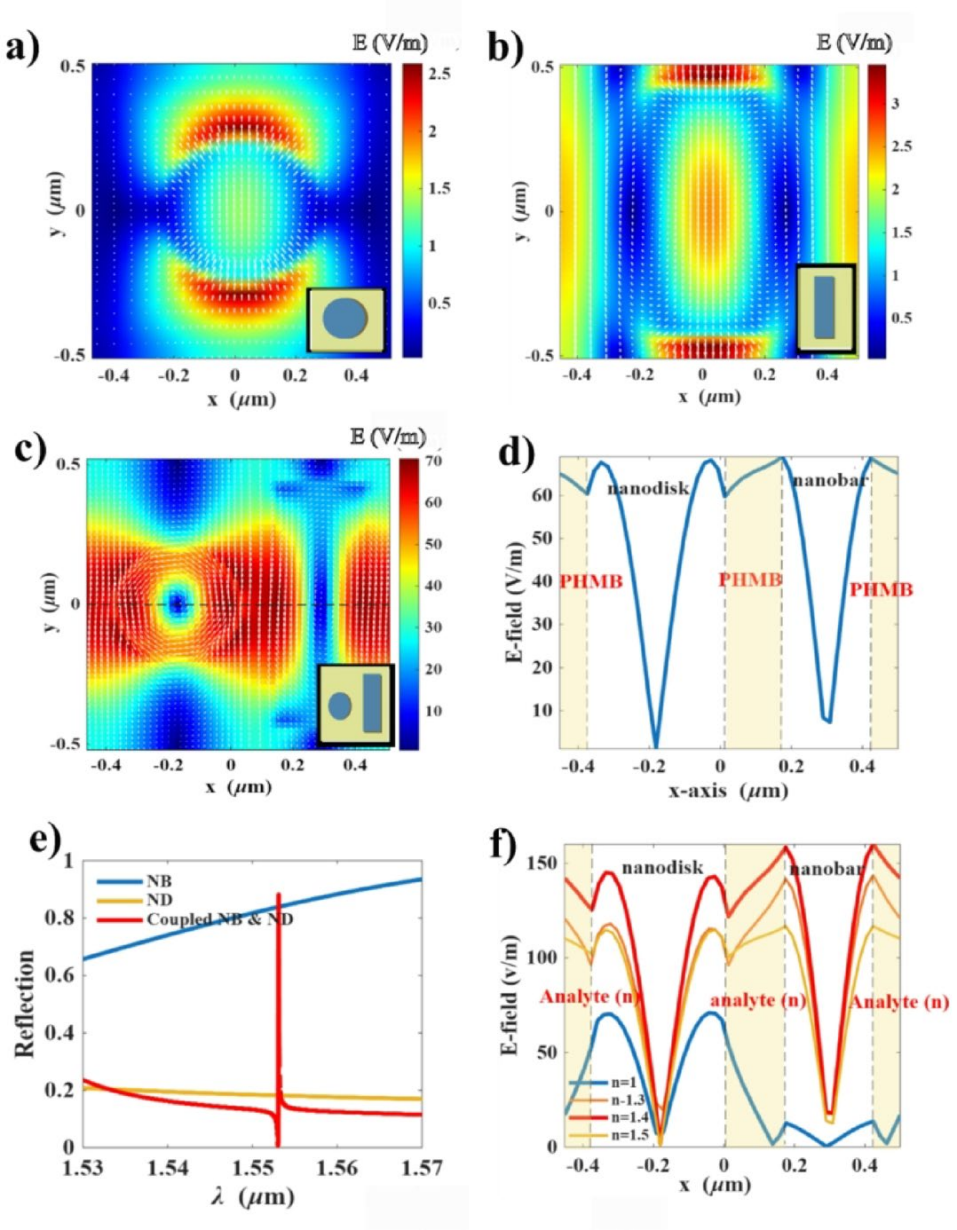
The two-dimensional E-field profiles across xy-plane of the three metasurface configurations infiltrated with PHMB polymer: **(a)** nanodisk (ND) only, **(b)** nanobar only (NB), and **(c)** coupled nanodisk and nanobar (coupled ND & NB) at λ = 1.553 μm. **(d)** line E-field profile along the dashed line in (c) for the initial geometry (g_1_= 165 nm). **(e)** normalized reflection spectra of the three configurations. **(f)** line E-field profile at the symmetry plane for different infiltrated refractive indices (*n* = 1.0, 1.3, 1.4, 1.54) using the optimized gap g_1_ = 162 nm. The increased field confinement with higher n confirms the role of PHMB in modifying the coupling mechanism.

In this section, we analyze the spectral response of the proposed design. Designing refractometric optical sensors requires generating a sharp spectral resonance that can be shifted by changes in the surrounding medium. Fano resonance is one of the most prominent phenomena characterized by sharp asymmetric spectral lines with distinct peaks and dips. This behavior can be achieved by inducing coupling between a bright mode—directly excited by the incident light—and a dark mode—excited under specific asymmetry conditions. Coupling of two types of resonators, nanodisk (ND) and nanobar (NB), has been proven to generate a Fano resonance in the reflection spectra^26^. However, the presence of infiltrated polymer alters the coupling mechanism. We investigate the electric field profiles (E-field) across three metasurface configurations infiltrated with PHMB polymer, nanodisk (ND) only, nanobar (NB) only, and the coupled nanodisk and nanobar (coupled ND & NB) with broken symmetry in the gaps between the resonators. Figure 2a and b show the 2D electric field profiles in the xy-plane for both isolated configurations—ND and NB. It is observed that both configurations excite bright modes with high damping rates in total transmission (orange line in Fig. 2e) and reflection (blue line in Fig. 2e), respectively. However, when the two resonators are coupled with asymmetric gaps, the dark mode is excited, inducing circulating electric fields on each resonator with significant confinement within the PHMB-filled gap (Fig. 2c and d). This confinement results in the emergence of Fano resonance (red line in Fig. 2e). This is different from the case without PHMB as presented in previous work^26^. This discrepancy is addressed by investigating the line electric field profiles at the symmetry plane for the structure with g_1_=162 nm, for refractive indices (*n* = 1 (without PHMB), 1.3, 1.4, 1.54 (PHMB)), as shown in Fig. 2f. It is observed that in the case without PHMB (*n* = 1.0, blue line in Fig. 2f) a strong dark mode is excited inside the nanodisk, and the field is less confined in the gap between the resonators, while increasing the refractive index of the infiltrated analyte leads to enhanced field confinement in the PHMB layer. This represents a slight shift in the coupling mechanism, which is of pivotal importance for CO_2_ sensing.

Moreover, this coupling results in significant suppression of the structure losses. The single resonators metasurfaces (periodic nanodisks and periodic nanobars) exhibit dramatic losses presented as broad reflection spectra as shown in Fig. 2e. These losses are referred to as radiative losses. However, by coupling both resonators, the non-radiative losses of the Fano resonant excited mode are dominant. The Fano resonance emerges due to the excitation of the dark mode, which introduces non-radiative losses and suppresses the radiative leakage from the nanodisk and nanobar. The measured Q-factor of ~ 8 × 10⁴ indicates that non-radiative losses (e.g., material absorption in silicon and residual scattering from fabrication imperfections) dominate over radiative losses. PHMB itself has negligible absorption at 1.55 μm, so it does not introduce additional non-radiative loss. Thus, the polymer infiltration primarily reduces radiative leakage, increasing Q-factor, while the non-radiative losses remain unchanged.

### Optimization of the gap between the resonators

**Fig. 3:**
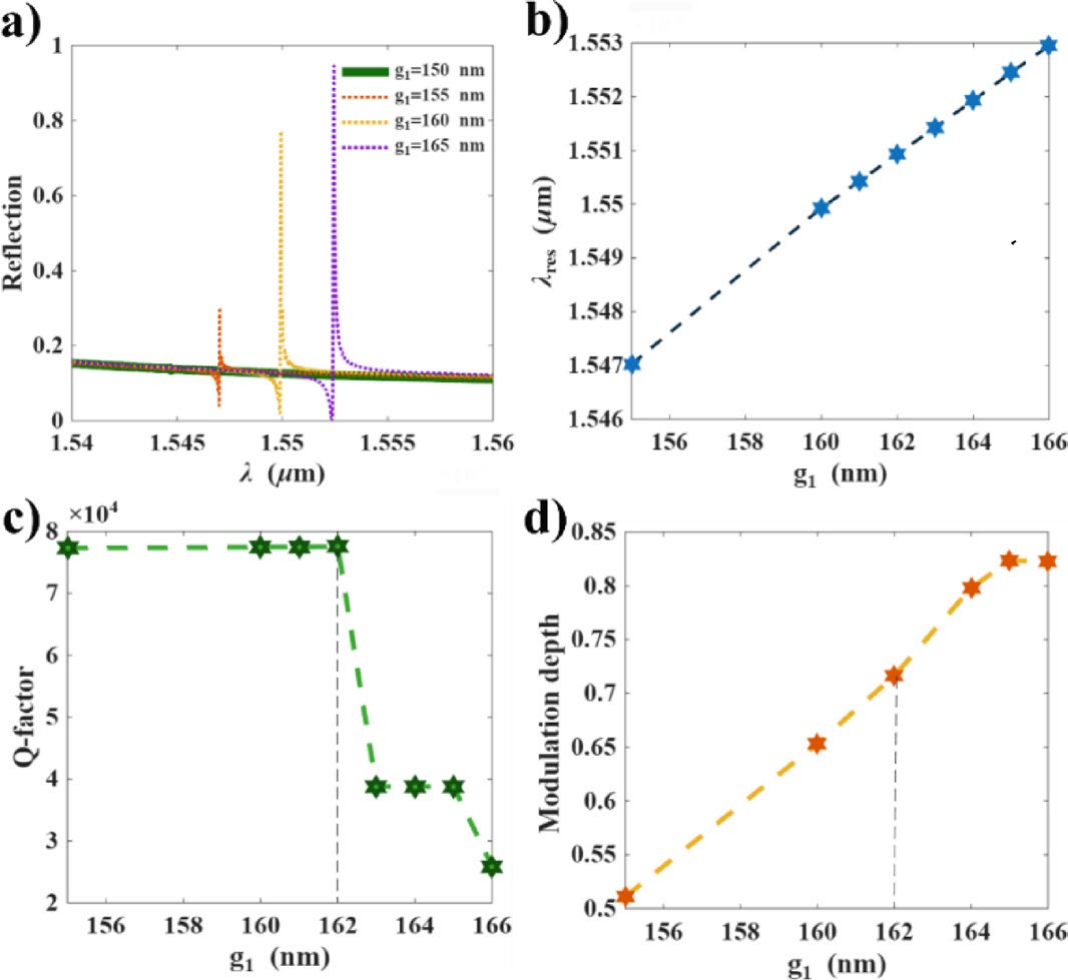
The effect of the first gap (g_1_) geometry on: **(a)** reflection spectra, **(b)** resonance wavelength, **(c)** Q-factor, and **(d)** modulation depth.

In this section, we optimize the gap geometry (g_1_), while keeping (g_2_) fixed at 150 nm, to achieve the highest Q-factor with a good modulation depth in the normalized reflection spectra. We begin with the symmetry condition where (g_1_=150 nm), which verifies the absence of Fano resonance for this geometry (green line in Fig. 3a). Further, we increase (g_1_) and calculate the corresponding resonance wavelength (λ_res_), Q-factor, and modulation depth, as shown in Fig. 3. We observe that increasing (g_1_) results in a redshift of the resonance wavelength (Fig. 3a and b), while maintaining a significantly high Q-factor reaching 77,521 for (g_1_=162 nm). However, this high Q-factor is enhanced at the expense of modulation depth for values of (g_1_) smaller than 162 nm, depicted by dashed lines in Fig. 3c and d, where the modulation depth reaches 72%. This geometry (g_1_=162 nm) is selected for sensor design.

### CO_2_ Gas Sensing using Wavelength Interrogation Method

PHMB is a polymer that belongs to the guanidine family, possessing a basic guanidine functional groups. PHMB has been proven to be a highly sensitive and selective polymer to CO_2_ gas undergoing the following reaction:
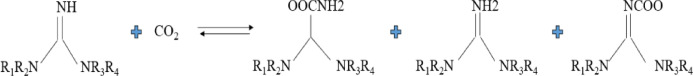


This reversible reaction easily occurs at room temperature and atmospheric pressure, resulting in a redistribution of the electron density within repeating units of the polymer. Consequently, the polarizability of PHMB changes due to the binding of CO_2_ molecules, forming a negatively charged carbamate ion^28^. This guanidine–carbamate interaction is the dominant mechanism underlying the CO₂ selectivity at the material level. In contrast, materials such as volatile organic compounds (VOC), lack a functional group that can interact with the guanidine group. In addition, PHMB polymer has been experimentally proven to be highly selective to CO_2_ over other gases such as nitrogen, Argon and hydrogen^36^. However, the key limitation is its cross-sensitivity to humidity and temperature^37^. Therefore, the humidity and temperature calibration and compensation are important for precise CO_2_ sensing. This can be achieved through developing analytical modelling or using additional sensors for humidity and temperature.

The intrinsic chemical stability of the guanidine group in PHMB is excellent under normal operating conditions. The polymer is known to be stable against light and heat, ensuring a stable baseline over extended periods. Fortunately, PHMB has experimentally demonstrated a consistent linear sensitivity behaviour under constant temperature and relative humidity^37^. Moreover, PHMB repeatability has been examined in various literatures showing excellent repeatability after purging the N_2_ gas, with acceptable standard deviation $\:\pm\:5.3$^38^.

In ref^38^, the refractive index of PHMB layer has been measured using Filmetrics reflectometer demonstrating a refractive index of 1.56 at wavelength of 632 nm. Using Cauchy model fit, the study extrapolated the PHMB refractive index at a wavelength of 1.55 μm to be *n* = 1.54, based on the negligible absorption across the measuring wavelength range 632–1550 nm^39^. The study reported the sensitivity of a micro-ring at various CO_2_ concentrations. For zero CO₂ concentration, the refractive index of PHMB is taken as *n* = 1.55^40^. In ref^41^, the reported data are used to infer the change of the refractive index of PHMB layer upon CO_2_ absorption. However, PHMB as a member of guanidine family is rich in receptor sites and possesses high capacity for CO_2_ adsorption reaching 2.4 mmol/g for poly(guanidine ethyl methacrylate) (PGEMA)^42^. These characteristics are affected by the environmental conditions, specifically the humidity, pressure and temperature^37^. Therefore, developing an adsorption isotherm model of CO_2_ on PHMB layer is predicted to provide comprehensive information including the adsorption uptake and saturation levels at specified temperature and pressure. The adsorption uptake directly leads to a change in the polymer index of refraction. A multilayer model with saturation is appropriate equilibrium isotherm for a thick polymer, describing the physicochemical parameters^43,44^. Meanwhile, the refractive indices and thicknesses of PHMB with various CO_2_ concentrations can be measured using a spectrophotometer, followed by fitting the transmittance−reflectance to the Cauchy model for dielectric materials, using a modified Levenberg−Marquardt method^45^. The CO_2_ uptake described by the isotherm model and the available refractive index data can be correlated and linearly calibrated, providing refractive index information. Further, the obtained refractive indices are used as inputs in the electromagnetic simulations of metasurface exhibiting spectral shift upon changing the index of refraction of the mounted polymer. In this work, we use the data referenced in^41^, inferring the decrease in the refractive index with the small changes in CO_2_ concentrations, as depicted in Table 1.

**Fig. 4:**
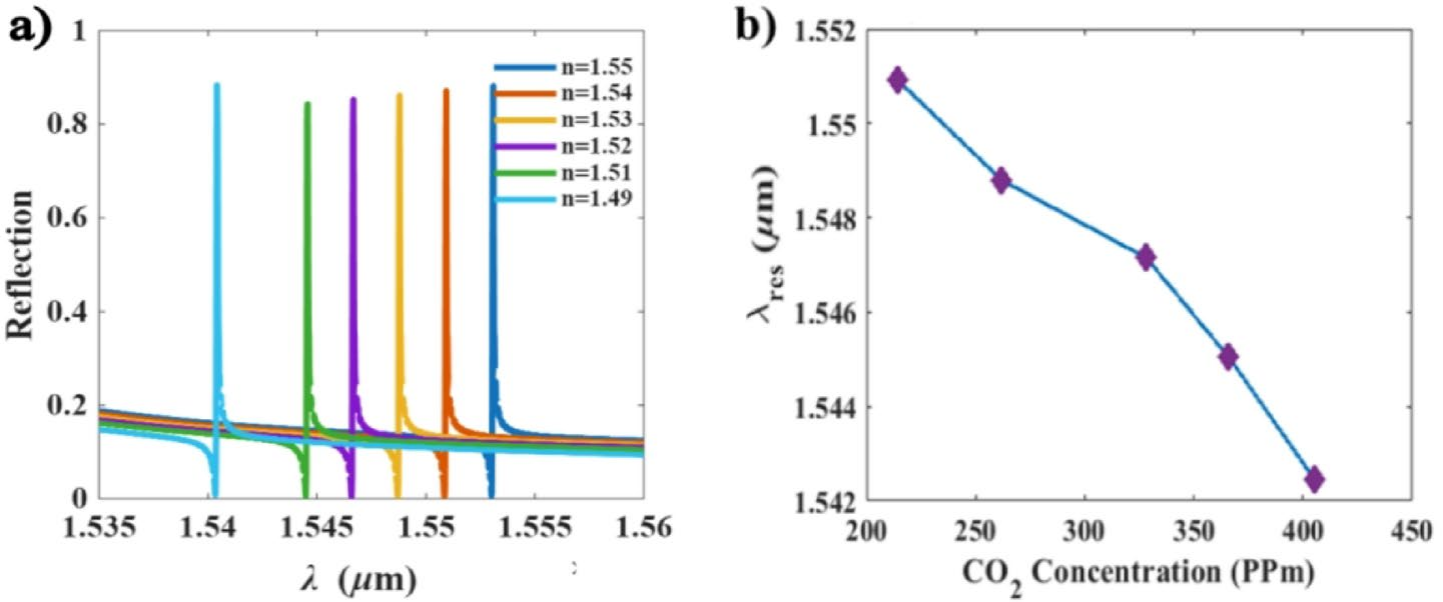
Sensitivity of the design at different CO_2_ concentrations: **(a)** reflection spectra for various refractive indices as a function of atmospheric CO_2_ concentration, **(b)** resonance wavelength at different CO_2_ concentrations.

We investigate the sensitivity of the sensor at various CO_2_ concentration. This results in blue shifts in the resonance wavelength due to the decrease in the refractive index, as shown in Fig. 4a and b. The change of refractive indices is not perfectly linear with the CO_2_ concentrations. Specifically, the concentration of 215 ppm has been presented as a threshold of the PHMB refractive index change. Therefore, the sensitivity was calculated over the concentration range from 215 ppm to 262 ppm, yielding a remarkable sensitivity of 45 pm/ppm. Based on this calculation, a concentration difference of 47 ppm corresponds to a refractive-index sensitivity of 212 nm/RIU. Furthermore, the limit of detection (LOD) of the device was calculated to be $\:9.34\times\:{10}^{-5}$RIU.

**Table 1:** Relationship between atmospheric CO₂ concentration (ppm) and the refractive index of PHMB.

PHMB refractive index (*n*)	Atmospheric CO_2_ concentration (ppm)
**1.55**	0
**1.54**	215
**1.53**	262
**1.52**	328
**1.51**	366
**1.49**	434

### Sensitivity at various PHMB thicknesses

**Fig. 5:**
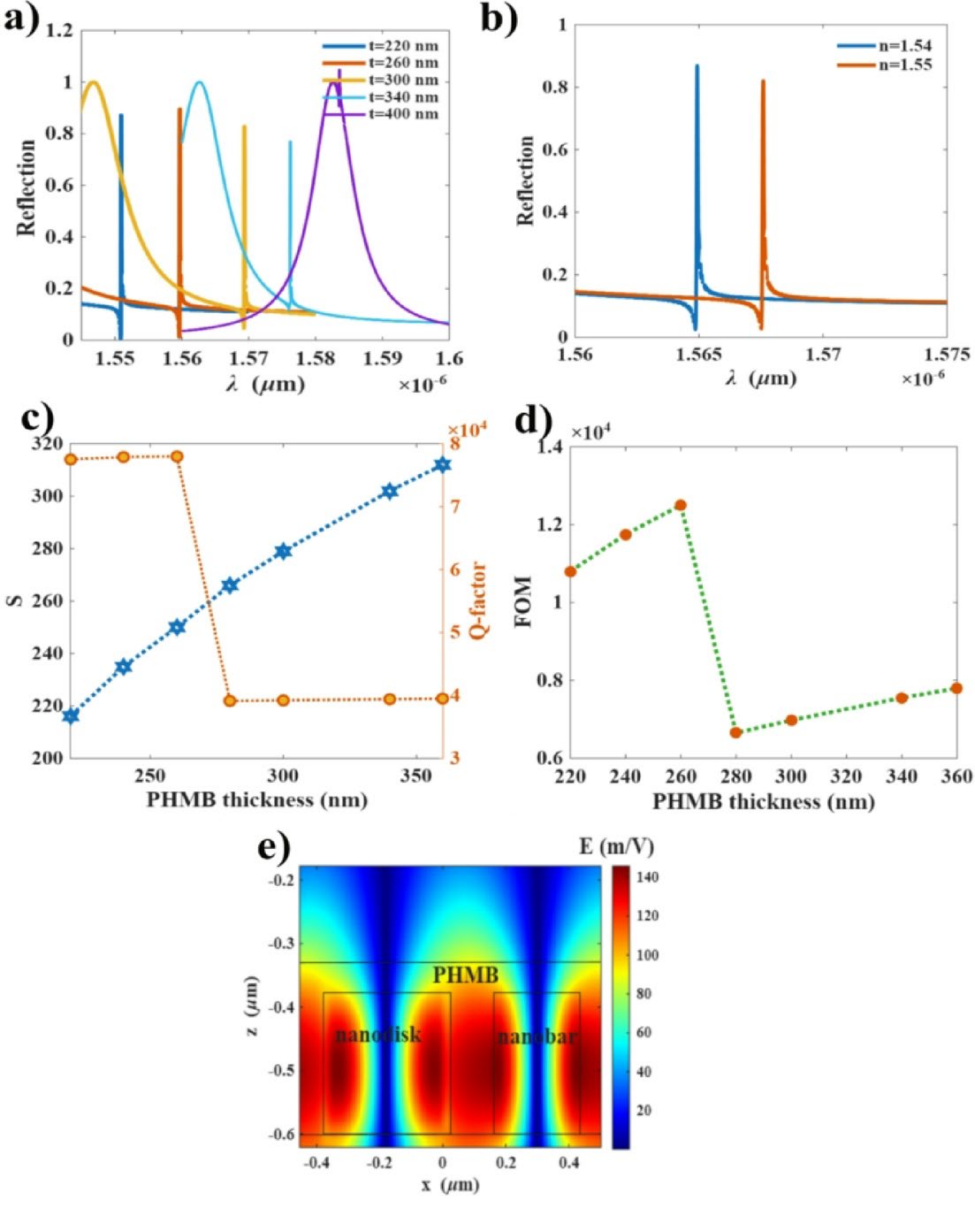
Effect of PHMB thickness on sensor sensitivity performance: **(a)** reflection spectra for different PHMB thicknesses, **(b)** reflection spectra at a thickness of 280 nm for two refractive indices, **(c)** sensitivity (S) and Q-factor as functions of PHMB thickness in the range of 220–360 nm, **(d)** FOM across the same thickness range. (e) Vertical cross-section of the electric field profile at the middle of the structure, thickness 270 nm.

In this section, we study the effect of increasing PHMB thickness on sensitivity performance. It is observed that increasing the PHMB thickness up to 380 nm results in a red shift of the Fano resonance. However, further increasing the thickness to 400 nm leads to disappearance of Fano resonance, as shown in Fig. 5a. Moreover, the sensitivity of different thicknesses is evaluated by calculating the spectral shift between the PHMB layer without CO_2_ adsorption (*n* = 1.55) and that with low CO_2_ concentration adsorption (*n* = 1.54), as illustrated in Fig. 5b. Increasing the thickness enhances the sensitivity, reaching a maximum value of 312 nm/RIU at a thickness of 380 nm. However, the smaller thicknesses (220 nm to 260 nm) exhibit significantly high Q-factor, reaching approximately 8$\:\:\times\:{10}^{4}$, as shown in Fig. 5c. The higher thicknesses show a drop in Q-factor as the field confinement degrades due to Fano mode leakage into the broad mode. This indicates a trade-off between sensitivity and Q-factor. The overall sensor performance is evaluated by calculating the FOM using Eq. (3), which reaches 12,500 RIU^− 1^ corresponding to sensitivity (250 nm/RIU) and FWHM of 0.02 nm for a thickness of 260 nm. In addition, the LOD is enhanced down to $\:8\times\:{10}^{-5}\:$RIU.

This enhancement is attributed to the increased interaction area between the evanescent field and the sensing material as illustrated for the structure with a thickness 270 nm in Fig. 5e. However, exceeding a thickness of 260 nm results in the appearance of a broad spectral line close to the Fano resonance as shown in Fig. 5a (yellow, blue and purple curves). As the thickness increases, the two modes become closer and merge at a thickness of 400 nm, resulting in almost complete disappearance of Fano resonance as depicted in Fig. 5a (purple curve).

The device FOM value represents an outstanding performance, as compared with the state-of-the art sensor platforms. A comparison of FOM of various platforms is presented in Table 2.

**Fig. 6:**
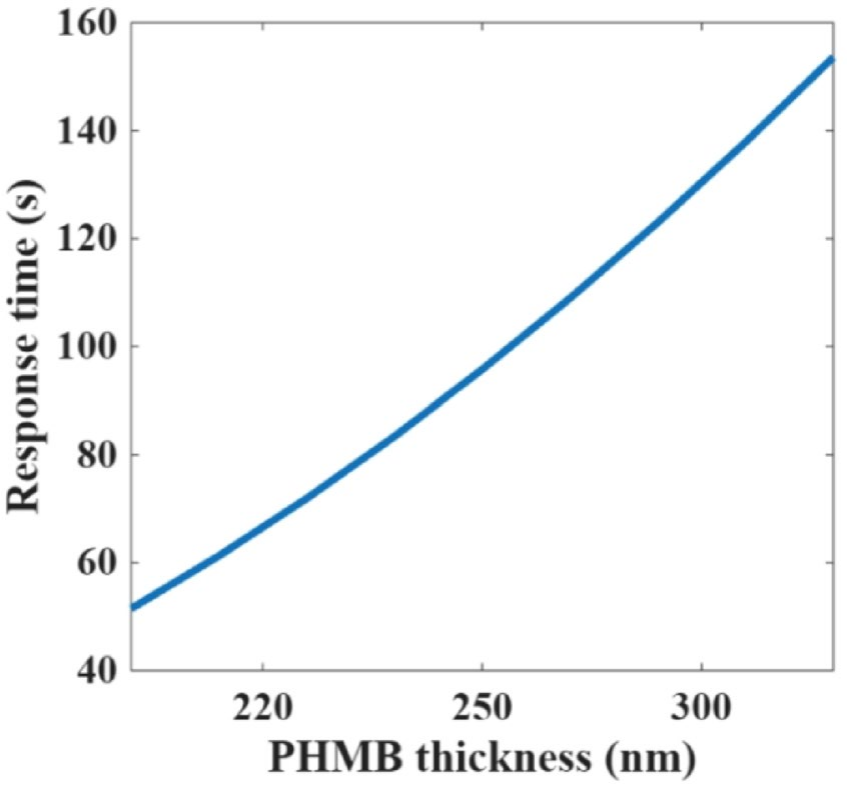
Sensing response time for various PHMB thicknesses at constant concentration of CO_2_ (5000 ppm).

Although increasing the PHMB thickness enhances the sensitivity, it is expected to increase the response time to allow CO_2_ to diffuse into the polymer layer and undergo the adsorption or desorption reaction. For the hybrid metamaterial-PEI polymer absorber platform, the steady state response that indicates the maximum signal output of complete adsorption/desorption process of PEI -a member of guanidine polymers as PHMB- of thickness of 300 nm is close to 2 min for various CO_2_ concentrations up to 177 ppm^34^. After that, the polymer becomes saturated and the sensor differential absorption spectrum slope declines. For the microring resonator with infiltrated PHMB, the response time is around one minute for thickness of 240 nm^38^. Moreover, the saturation ppm level of PHMB of thickness of 240 nm is significantly higher (5000 ppm) than saturation of PEI of thickness of 300 nm (177 ppm). However, the saturation ppm level of both polymers can be enhanced by increasing the polymer thickness that enhances the adsorption capacity. The relation between the response time and the polymer thickness can be evaluated using Fickian diffusion (characteristic time $\:\tau\:=\frac{{L}^{2}}{D}$), where L is the polymer thickness and D is the diffusivity coefficient. The diffusivity D of CO_2_ into PHMB can be modelled using the Fickian diffusion coefficient. The analytical models show that the diffusivity is dependent on particle concentration (CO_2_) and the type of the environment, attractive or repulsive. PHMB is an attractive environment because it interacts with the CO_2_ molecules resulting in reducing diffusivity due to the interaction with shallow sites^46^. Based on the experimental results of the micro ring resonator at a CO₂ concentration of 5000 ppm, the response time of around one minute for a complete adsorption/desorption cycle yields a diffusivity on the order of 9.4 × 10⁻¹² cm²/s. The calculated response time as a function of the PHMB thickness is shown in Fig. 6. However, the major challenge of high polymer thickness is the difficulty to remove all adsorbed CO_2_ molecules upon purging the N_2_ gas, and the spectrum doesn’t return to its baseline for repeated measurements.

To the best of our knowledge, the hybrid metasurface-PHMB design approach exhibits superior sensitivity/selectivity performance to CO_2_ as compared to other sensors. For instance, various high-sensitivity approaches such as metasurfaces^15^, Mach-Zehnder Interferometer (MZI)^47^ and metal-insulator plasmonic waveguide^48^, are tailored to match the gas absorption fingerprints. However, these approaches suffer from the poor gas-light interaction. Other approaches use different types of adsorbent materials such as the metal organic frameworks (MOF). These organic materials are suitable for sensing large molecules including the volatile organic compounds (VOC)^49^. Alternatively, the approaches that use PHMB such as Au-disk/TiO2- cylinder/Au-film metasurface coated by (PHMB) film demonstrate exceptional sensitivity (109 pm/ppm), at operational wavelengths between 1.027 and 1.060 μm for the CO_2_ concentration range (200–600 ppm)^33^. Despite that our sensor exhibits a lower sensitivity of 45 pm/ppm over a similar, though not identical, concentration range (0–434 ppm), the superiority of our design stems from utilization of two crucial aspects. First, the use of all-silicon dielectric material offers the advantage of low losses, in contrast to the intrinsic losses of their metallic counterparts. Such low losses yield an outstanding Q-factor of ~ 8$\:\times\:{10}^{4}$ and a FOM of 12,500 RIU^− 1^, respectively, which together enable high-sensitivity precision. Second, our design works at the telecommunication wavelength of 1.55 μm, which plays the central role in PIC systems. This makes its integration with photonic components feasible and compact.

Despite the significant Q-factor of the simulated design, the experimental verification is expected to reduce it due to fabrication imperfection and geometrical perturbation^50^. In addition, the minute error in fabrication geometry from g_1_=162 nm to g_1_= 163 nm results in reducing the Q-factor to approximately half its value, as shown in Fig. 3c. Moreover, at the edges of metasurface, the coherent oscillation of dark modes breaks leading to a strong scattering in free space and resulting in broadening of the spectrum. The perturbation due to edges can be minimized by increasing the metasurface array size until saturation is reached, beyond which no further Q-factor enhancement is observed. In ref^50^, saturation has been achieved using 90,000 unit cell. In addition, the surface states arisen in Si during etching process provide additional source of losses. The scattering at edges, the surface states and geometric perturbation contribute to a considerable degrade in Q-factor, form 30,000 to 483 for coupled nanoring and nanobar metasurface. Nevertheless, the dielectric Si is an excellent candidate in designing optical devices due to mature fabrication process and the compatibility with modern CMOS technologies leveraging its high refractive index. This high refractive index accompanied by the zero losses in near IR potentially provides exceptional stability of Fano resonance over time. Thanks to the electron beam lithography (EBL), the CMOS-compatibility of Si-based devices with high resolution, reliable repeatability and scalability to mass manufacturing are achievable^43^. The EBL is the predominant technique for dielectric nanofabrication that precisely sculps the electron beam resist at the nanoscale based on a computer-generated pattern. In addition, the PHMB layer repeatability is determined by returning the spectrum to its baseline upon purging of N_2_ gas. Ref^38^ defined the standard deviation in resonance shifts after CO_2_ adsorption recovery to be $\:\pm\:0.071\:$pm. In Table 2, we present a comparative analysis of our hybrid metasurface-PHMB design with other reported gas sensors.

**Table 2:** Comparative analysis of various gas sensor approaches.

Sensor design	Sensing material	Selectivity approach	Working wavelength	Sensitivity	Q-factor			FOM	Ref.
Multiple‑Mode Bowtie Cavities	Glucose			1500 nm/RIU				50 RIU^− 1^	^10^
Plasmonic photonic crystal fiber	temperature	Energy transformation from the core-guided mode to the (SPP) mode.	712 nm	5200 pm/^0^C	237			1.73/^0^C	^14^
Fiber Fabry-Perot Interferometer Based on PEI/Poly (Vinyl Alcohol) Coating	CO_2_	Adsorbent polymer (PEI/Poly (Vinyl Alcohol)	1.527 μm	450 nm/RIU	Limited				^29^
Au-disk/TiO2- cylinder/Au-film metasurface coated by (PHMB) film	CO_2_	Adsorbent polymer (PHMB)	1.33 μm 1.04 μm	42.57 pm/ppm 109.25 pm/ppm	Limited				^33^
Silicon microring resonator (MRR) integrated with PHMB		Adsorbent polymer (PHMB)	1.55 μm	3.$\:54\times\:{10}^{-3}$pm/ppm	20,000			$\:{46\times\:10}^{-5}$ ppm^− 1^	^30^
Plasmonic silicon Mach-Zehnder interferometer (MZI).		Absorption peaks match gas absorption fingerprint	5.300 μm	16,000 nm/RIU				114 RIU^− 1^	^47^
Metal-insulator (MI) plasmonic waveguide in the mid infrared range and utilizing a Mach-Zehnder Interferometer (MZI)		Absorption peaks match gas absorption fingerprint	4.500 μm	10,000 nm/RIU				363 RIU^− 1^	^48^
MIM waveguide coupled Square Convex Ring Resonator with metallic baffle	Refractive index			1120 nm/RIU				2.68$\:\times\:{10}^{5}$	^51^
Hybrid Photonic Cavity with Metal-Organic Framework (MOF) Coatings	Volatile organic compound (VOC)	Adsorbent polymer (MOF)	1.55 μm	5000 nm/RIU	23,000			14,839 RIU^− 1^	^52^
Coupled nanodisk and nanobar resonators integrated with MOF	Ethanol	Adsorbent polymer (MOF)	1.38 μm	320 nm/RIU	6,545			1518 RIU^− 1^	^49^
Proposed Hybrid Metasurface-PHMB	CO_2_	Adsorbent polymer (PHMB)	1.55 μm	250 nm/RIU	~ 8$\:\times\:{10}^{4}$			12,500 RIU^− 1^ (2.25 ppm^− 1^)	Our work

### Proposed fabrication procedure

**Fig. 7:**
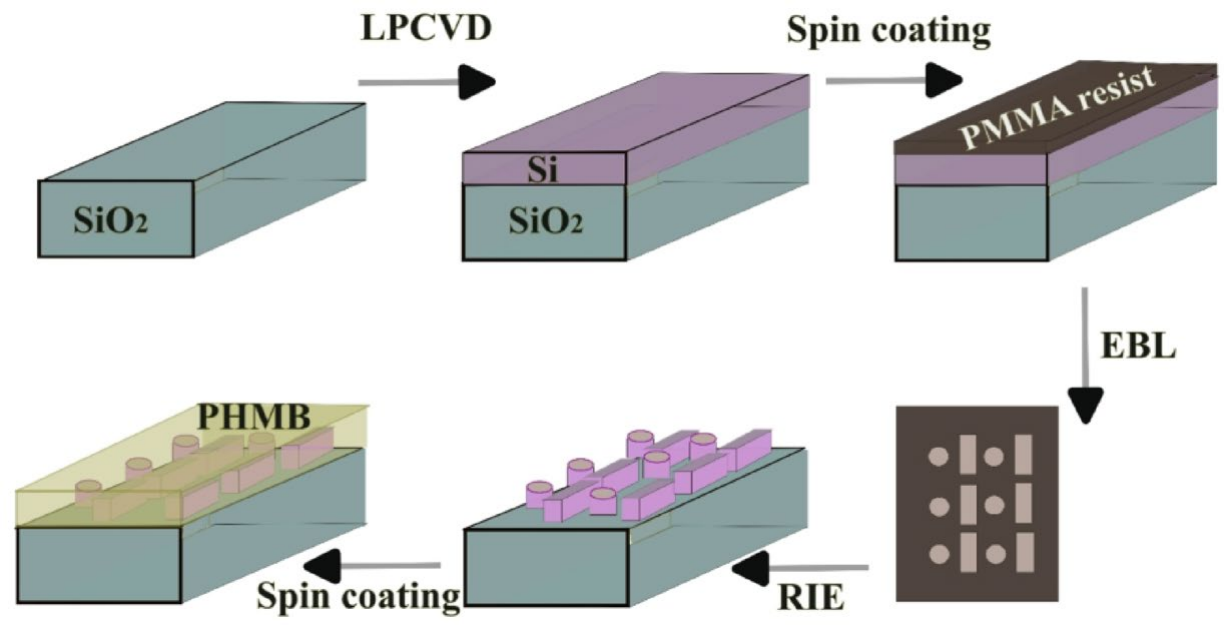
fabrication steps of the hybrid metasurface CO_2_ sensor using electron-beam lithography (EBL): initially, a Si layer is deposited on a SiO_2_ substrate using low pressure chemical vapor deposition (PLCVD), followed by spin coating of electron beam resist (PMMA), direct writing of the metasurface pattern by EBL, reactive ion etching (RIE), and finally, PHMB deposition.

Metasurface designs benefit from the ease of fabrication using conventional techniques^53^, including atomic layer deposition (ALD)^54^, photolithography^55^, and electron-beam lithography (EBL)^56^. The coupled nanodisk and nanobar metasurface design on a substrate has previously been fabricated using EBL, as reported in Ref^56^. The proposed fabrication steps of the coupled resonators hybridized with PHMB polymer are illustrated in Fig. 7. Initially, a Si layer, serving as a functional metasurface material, is deposited on a SiO_2_ substrate using low-pressure chemical vapor deposition (LPCVD). Subsequently, an electron-beam-sensitive resist layer, such as PMMA, is applied by spin coating. The metasurface pattern is then directly written onto the PMMA resist using EBL. A chromium dissipation layer is deposited on top of the PMMA layer to prevent charge accumulation during exposure. Next, an appropriate reactive ion etching (RIE) process is employed to transfer the imprinted metasurface pattern into the Si layer. Finally, a PHMB layer is deposited by spin coating, and its thickness is controlled by adjusting the spin speed and the solution concentration^38^.

## Conclusion

In this work, we report a selective CO_2_ refractometric sensor based on a hybrid metasurface-PHMB polymer design. PHMB polymer serves as an excellent functional material that selectively interacts with CO_2_ molecules with the advantage of reversible reactions under atmospheric temperature and pressure. The proposed sensor operates at the telecommunication wavelength of 1.55 μm. The sensor demonstrates outstanding sensitivities in the range of 216–312 nm/RIU, accompanied by remarkable Q-factors of up to 8$\:\times\:{10}^{4}$ for small PHMB thicknesses, resulting in a high FOM of 12,500. This distinct performance combined with low cost, the ease of fabrication, and CMOS compatibility, highlights the design as a strong candidate for practical sensing applications. Furthermore, its operation at telecommunication wavelengths makes it highly suitable for integration into photonic integrated circuits. In future work, this design can be developed for thermal and electrical tunability purposes. For instance, PHMB has been reported in a dual-mode microring resonator for the relative humidity sensing with temperature compensation, demonstrating thermal sensitivity^37^. This study suggests that PHMB can act as a thermally active material and hence, a thermally tunable Fano resonant metasurface can be achieved. Electrical tunability can be achieved by integrating our Fano resonant device with a graphene layer, whose Fermi energy is controlled through electrostatic gating^57^.

## Materials and methods

The finite-difference time-domain (FDTD) method using Lumerical software was used to simulate the optical response of the proposed structures to incident plane waves.

## Data Availability

The datasets used and/or analysed during the current study are available from the corresponding author on reasonable request.
